# Advancements in bacteriophage therapies and delivery for bacterial infection

**DOI:** 10.1039/d2ma00980c

**Published:** 2023-01-31

**Authors:** Hannah A. Durr, Nic D. Leipzig

**Affiliations:** a Department of Chemical, Biomolecular, and Corrosion Engineering, University of Akron Ohio 44325 USA nl21@uakron.edu; b Department of Integrated Biosciences, University of Akron Ohio 44325 USA

## Abstract

Having co-evolved with bacteria over hundreds of millions of years, bacteriophage are effective killers of specific bacterial hosts. Therefore, phage therapies for infection are a promising treatment avenue, can provide a solution for antibiotic resistant bacterial infections, and have specified targeting of infectious bacteria while allowing the natural microbiome to survive which systemic antibiotics often wipe out. Many phages have well studied genomes that can be modified to change target, widen target range, or change mode of action of killing bacterial hosts. Phage delivery can also be designed to increase efficacy of treatment, including encapsulation and delivery *via* biopolymers. Increased research into phage potential for therapies can allow new avenues to develop to treat a larger range of infections.

## Abstract 2

Graphical representation of phage natural replication, molecular modification applications including CRISPR/Cas9, and delivery applications including hydrogels and liposome encapsulation for treatment of bacterial infections.

## Graphical abstract

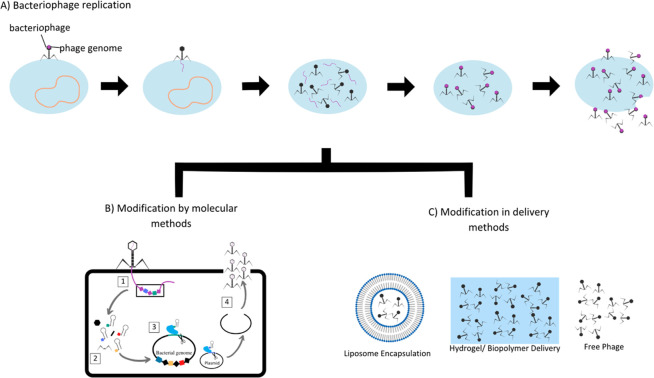

## Introduction

Bacteriophage, or phage, are bacteria-specific viruses with an outer protein capsid surrounding phage genetic material, and in some cases, filamentous tails. They are highly abundant and variable, and play important roles in impacting microbial ecology.^1^ Phage have co-evolved with bacteria over hundreds of millions of years, and selectively bind to and infect their target hosts, leading to their capability to influence the population dynamics of multi-strain microbial populations by targeted lysis. Additionally, most are highly stable long-term if kept in a non-hostile environment, only being broken down in UV light or damaged by physical abrasion or exposure to certain chemicals with a few exceptions. Phage genomes are small and relatively simple, allowing engineering *via* synthetic biology approaches to deliver small molecules to invading infection, expand or narrow the target of phage therapies, or be utilized in combination with biomaterials in wound healing technologies. The goal of this review is to describe various avenues of phage therapy for the treatment of infections, including chronic and multidrug resistant populations of bacteria. Specific focus is placed on delivery methods for phage and both the advantages and disadvantages of selected strategies.

## History

Bacteriophages were discovered in 1915 by William Twort and later their identity was confirmed in 1917 by Felix D’Herelle, whom documented their potential to kill bacteria.^2^ However, in western countries most focus on their use as therapeutic agents was lost with the discovery and widespread adoption of chemical-based small molecule antibiotics in the 1940s, which could be easily administered comparably to phage therapies.^3^ An overall lack of understanding and poor scientific design contributed to the skepticism by western countries to utilize phage therapies, with many referential old Soviet experiments designed with missing placebos, inaccurate or missing phage preparation, unequal or missing numbers of patients in experimental and control groups, or in some cases, no control groups reported at all.^2^ Russian and Georgia (both members of the former USSR) have continued research into phage therapies and have developed approaches to isolate and use phage to treat diseases.^2,3^ Although phage therapies have been somewhat abandoned in western countries, the evolution of drug-resistant to multi-drug resistant bacterial infections has increased recent interest in phage therapy as an alternative to traditional antibiotic treatments.

Phage therapies have been described in literature going back almost 100 years, with reports from French and British literature in the 1920s noting successful treatment of typhus caused by rickettsia bacterial infections, using intravenous (IV) administered phages.^4–6^ Knouf *et al.* described in 1946 a 10 year study on the use of phage therapies to treat typhoid at the Communicable Disease Unit at the Los Angeles County General Hospital. They reported out of 56 patients treated with phage, the mortality rate was 5% compared to the typical mortality rate of typhoid fever of 14% without the use of phage therapy in the same hospital.^7^ In France, the IV route of administration was highly reported on, especially for use against *Staphylococcus* species infections as described by Vieu of the Bacteriophages Service of Pasteur Institute in 1961.^8^ Vieu also specifies three key components to these phage treatments: (i) exclusively virulent phages were used, (ii) the quality of the media used to produce phages for IV use was important and that the media best tolerated contained the least amount of large protein molecules, and (iii) if phage therapy was long-term enough to induce antibody production, then thought should be given before adding an additional phage that may cross-react serologically.^8,9^

**Fig. 1:**
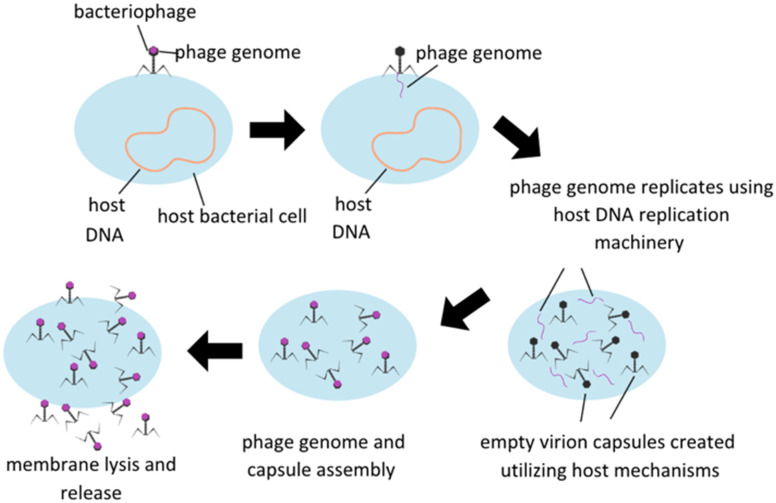
Lytic bacteriophage replication schematic. Modified from Düzgünes *et al.* (2021).

Treatment utilizing phage is based on the fundamental nature of lytic phages, which specifically infect live bacteria, replicate themselves to maximum capacity, and then spread the phage progeny *via* lysis of the bacterial membrane, subsequently killing the host (Fig. 1). Phage infection is reliant on surface receptors on bacterial membranes, which have the advantage of limiting phage infectability to bacterial hosts only. Some other potential benefits of utilizing phage therapy include reduction in interactions between antibiotics and other drugs in an *in vivo* system, avoiding antibiotic toxicities, and ability to retain activity in infections developing a biofilm which are a protective accumulation of extracellular polymeric substances (EPS) secreted by a group colonization of bacteria that serve to protect the bacteria's integrity and ability to continue to proliferate. Phages are designed to seek and destroy biofilms *via* various mechanisms, including degradation of the extracellular matrix, penetration of the biofilm, and infection of bacteria within the biofilm. Once self-injected into a host bacterium, phage genome can stimulate the host to produce enzymes to degrade EPS. Upon degradation, phage penetration, replication, and lysis of other bacterial hosts can be facilitated.^10^ There are some concerns, however, including potential genetic contamination of the microbiome leading to virulence factors or antibiotic resistance, development of bacterial resistance to phage, and the implications of endotoxin release upon bacterial cell lysis and effect on the immune reaction. Host immune systems can also mount antibody based immune responses to phage pathogen associated molecular patterns (PAMPs) themselves, and this immune reaction will need to be carefully modulated to minimize immune reaction to phage therapies.^11^

The need for new antibacterial therapies is at crisis level as the occurrence of antibiotic resistant, multi-drug resistant, and extreme-drug-resistant strains of bacteria increases. Most strains of pathogens have become increasingly resistant to antibiotics, and many strains have become resistant to multiple antibiotics and chemotherapeutic agents.^12–14^ Some strains of bacteria have become resistant to almost all commonly available antibacterial agents, for example, methicillin-resistant *Staphylococcus aureus* (MRSA). MRSA, which is a major source of hospital-acquired infections, is resistant to methicillin, as well as aminoglycosides, macrolides, tetracycline, chloramphenicol, and lincosamides. Rare strains of MRSA have also developed resistance to vancomycin, which is currently one of the only effective antibiotics against it.^15^ Multidrug resistance in Gram negative bacteria is an increasingly concerning threat, which have an outer membrane with very low permeability and contain multiple antidrug efflux pumps to effectively evade most potential therapeutic agents.^16^ Multidrug resistance in bacteria is typically gained by acquiring a drug-resistance gene on a plasmid from certain streptomycetes, or various microorganisms in the environment.^15^

Upon infection, multidrug resistant bacteria can more easily colonize the host, producing a biofilm that is difficult to penetrate with traditional antibiotics. Over-accumulation of pathogens and biofilms can easily lead to bacteremia if the microbes move into the blood, septicemia if the microbes can grow in the blood, and eventually death. This is process is more easily accomplished in immunocompromised individuals.^16–18^

Current clinical applications using phage therapies are rare due to the long product development and approval processes for FDA clearance. This has led to limitations of phage therapies available for clinical treatment, and development of nonclinical applications to focuses in food safety, agricultural and clinical diagnostics.^19^ Multiple phage cocktail products have been FDA approved to treat processed foods or crop pathogens, including ListShield™ and LISTEX™ P1000 against *Listeria monocytogenes*, and Omnilytics' Agriphage™ which treats *Xanthomonas campestris pv. vesicatoria* and *Pseudomonas syringae pv. tomato*. Phage therapies are currently being developed against other pathogens, including *Escherichia coli* and *Salmonella enterica*.^20^ There are clinical therapies currently being tested for approval, however, they are typically still limited to animal studies. Reported studies have supported efficacy of phage therapies in animals against multiple pathogens, including *Pseudomonas aeruginosa*,^21^*Staphylococcus aureus*,^22^ vancomycin-resistant *Enterococcus faecium*,^23^*Clostridium difficile*,^24^ and *Klebsiella pneumoniae*.^25^ Some case studies have reported successful clinical outcomes in treating human infection, however the number of randomized control trials is too low to draw conclusions and to move toward FDA clearance in the immediate future.^26^

## Phage therapies for bacterial infection treatment

A benefit of phage therapies is the multiplicity in which phage can be administered to a host system. Topical, oral, and intravenous routes of administration allow for customization for treatment closer to the site of infection and can be used in combination with antibiotic treatment. Phage can be administered by a variety of methods, including liquid suspension or encapsulation in liposomes, or nano- or micro-particles.^27^ Benefits of encapsulation include increased phage stability and storage capacities with minimized loss of viability, which remediates a core problem with consistency of phage administration.

One potential response to the antibiotic-resistance crisis and treatment of bacterial infections could be application of phage therapies. Lytic phages have been shown to be able to selectively infect and lyse multi-drug resistant bacteria, providing an effective antibacterial response *in vitro* and *in vivo*. For example, Cao *et al.* showed efficacy of bacteriophage treatment against pneumonia induced by multidrug resistant *Klebsiella pneumoniae* in Swiss-Webster mice.^28^ The lytic phage against *K. pneumoniae* was isolated from sewage samples from the Zhongshan Hospital, characterized, and named Phage 1513. After initially confirming bactericidal abilities of Phage 1513 *in vitro*, mice were inoculated with a lethal dose of *K. pneumoniae* intranasally and a single dose of phage intranasally 2 hours after initial infection. Without treatment, 100% of mice died within 24 hours of infection. All groups with phage treatment had improved survival in a dose-dependent manner, the highest dose resulted in the greatest survival rate of 80%. Phage treatment was also shown the improve the pathology on the infected lung tissues, with only local, discrete lesions instead of intense alveolar abnormalities seen in the phosphate buffered-saline (PBS)-treated mice.^28^ Cao *et al.* and others have shown effective treatment of a multidrug resistant human pathogen *via* phage therapy.

Topical administrations of phage therapy have been reported in a variety of applications, including treatment of secondarily infected burn-mediated ulcers, infected diabetic foot ulcers, and other uncharacterized chronically infected wounds. A successful, commercially available wound dressing called Phagebioderm® was developed by the Eliava Institute in the country of Georgia, and targets *Pseudomonas aeruginosa*, *Staphylococcus aureus*, and *Streptococcus* species.^29^ This biodegradable polymer was embedded with a cocktail of lytic bacteriophages, antibiotic (ciprofloxacin), anesthetic (benzocaine 0.9 mg), and a wound healing agent (α-chymotrypsin) which helps resolve inflammation due to tissue injury and facilitate the repair process.^30^ Successful topical phage treatment defined by eradication of infection was achieved in 70% of patients with infected venous stasis ulcers or other drug-resistant infected ulcers in Tbilisi, Georgia.^29^ The topical application of phage therapies is based on multiplicity of infection (MOI) which defines the ratio of phage particles to bacterial cells. Efficacy of phage treatment is dependent primarily on phage concentration, increasing the MOI increases the success of therapy determined by a reduction in bacterial counts. In the case of burned epidermis, wound surfaces can be quickly colonized by bacteria, producing biofilms and potential resistance to one or multiple antibiotics.^31^ Kumari *et al.* infected a full-thickness burn wound with *Klebsiella pneumoniae* and compared treatments of silver nitrate, gentamicin, and phage Kpn5 in topical treatment of burn wound infections.^31^ All treatments were shown to effectively increase survival rate of infected animals, but Kpn5 was the most effective in antibacterial activity followed by 0.5% silver nitrate and 1000 mg gentamicin, respectively. Phage therapy was comparable in effectiveness to standard treatments, and the option for topical administration further solidifies its usefulness in treatment of wound surface infections. Treatment with phage with supplementation to traditional antibiotic treatment is a proven effective and viable treatment for a variety of bacterial infections, and likely will continue to be explored as the synergy of both phage and antibiotics appear beneficial to the wound healing environment.

McVay *et al.* further described phage therapy in a burn wound injury infected with *Pseudomonas aeruginosa* treated with a single dose of *P. aeruginosa* phage cocktail.^32^ Mice were thermally injured and 100 μL of inoculum was injected directly under the anterior end of the burn. Three different phages within the cocktail were administered by three different routes: intramuscular, subcutaneous, or intraperitoneal. The phage cocktail was shown to significantly decrease mortality of infected burned models without treatment (6% survival) compared to with phage treatment (22–87% survival based on mode of treatment). Subcutaneous was the least effective mode of administration, with 22% survival, intramuscular administration resulted in 28% survival, and intraperitoneal administration was the most successful treatment with 87% survival. This was suggested to be due to phage administered by the intraperitoneal method were delivered to the site of infection at a higher dose, delivered earlier, and delivered for a more sustained period of time than other routes. Phage cocktail was administered in all three routes in uninfected animals as a control, and all thermally injured non-infected mice treated with the phage cocktail survived, indicating the treatment was not toxic to traumatized mice.

Not only can phage therapies be effective *via* topical, intramuscular, subcutaneous, or intraperitoneal avenues, in liquid, spray, creams, gels, or powder form,^33^ veterinary medicine has explored the potential of oral administration. Due to their stable structure and easy genetic engineering potential, phage can be mutated to survive more effectively in gastrointestinal (GI) conditions, allowing for treatment of difficult GI infections. Phage have been encapsulated in base polymers, including alginate and pectin with oleic acid emulsification for oral administration to protect against the acidic environment of the host digestive system,^34^ but the selection of base materials can difficult because of certain constraints, such as the ability to be synthesized under mild environmental conditions, non-toxic, and environmentally friendly. Hydrophilic polymers are favorable for their stability in aqueous conditions, and phage encapsulation for treatment of low pH environments is necessary specifically to shield from protease activity and low pH of 1.6 in parts of the GI system. Non-encapsulated phage has been shown the become fully inactivated at pH 1.6 or with exposure to pepsin (0.5 mg mL^−1^) after 10 minutes.^34^ Nobrega *et al.* designed a non-encapsulation method of administration of phage, by genetically engineering the *E. coli* phage T7 to express the *E. coli* outer membrane phosphoporin protein E, which allows the natural bacteria resistance to the extreme pH environment of the gastrointestinal tract (GIT).^35^ The mutation did not affect the phage's ability to replicate or infect, and mutated T7 was shown to improve viability of T7 in a larger range of temperatures and acidity. Due to the well-known nature of T7 genetics, adjustments for improved efficacy are easily approachable, and show potential for application against other GIT bacterial infections. The potential for phage therapies within oral routes of administration was also explored against *Vibrio cholera* in rabbits by Abhishek *et al.* A cocktail of five lytic vibriophages was administered orally by gastric tube to New Zealand rabbits infected with *V. cholerae*, and bacterial counts were taken *via* fecal sheds before and after treatment with phage.^36^ Animals administered the phage cocktail 6 hours after the initial infection shed 100 fold lesser numbers of *V. cholerae* at the 60th hour compared to the 12th hour. Considering the current concern of cholera in developing countries, the re-emergence in many locales it was previously eradicated in, and the readily available transmission *via* water, an effective treatment against cholera could be of great benefit to humankind.

Intravenous (IV) administration of phage therapies has been described in various applications over the last century as well. Newer applications include a Korean drug (SAL200) based on a recombinant form of the phage endolysin SAL-1 to treat antibiotic-resistant staphylococcal infections. Jun *et al.* reported both short term stability of SAL200 (1 week) in monkeys, and single administration tolerance and pharmacokinetics in healthy human males.^37^ No serious adverse effects were observed through either study, with appropriate half-life and systemic clearing in monkeys, and no risks identified based on evaluation of vital signs, ECG, and physical measurements or remarkable changes in serum chemistry, hematology, or urinalysis for human subjects. These studies solidify the safety of utilizing this IV phage based therapies and provide a potential treatment avenue for difficult-to-treat antibiotic-resistant staphylococcal infections.^37,38^ Safety and efficacy of treating antibiotic-resistant staphylococcal infections in *in vivo* models has continued to be confirmed in studies throughout the last decade.^39^ Phage therapies have shown promise in specific severe infection cases in humans, including eradication of a multi-drug-resistant strain of *Acinetobacter baumannii* in a pancreatic pseudocyst of a 68 year-old diabetic man with necrotizing pancreatitis.^40^

## Phage-antibiotic synergy

Phage-antibiotic synergy (PAS) is an observed phenomenon in which sub-lethal concentrations of certain antibiotics can stimulate host bacterial cell production of some virulent phage to help fight off infections. Reports suggest that combination of phage therapies with low concentrations of designated antibiotics could provide a potential avenue of treatment for difficult to treat bacterial infections, including antibiotic resistant infections.^41,42^ This phenomenon has been observed in T4-like phages with β-lactam, quinolone, and mitomycin C antibiotics, as well as an increase in phage ΦMFP production by more than 7-fold by a uropathogenic *Escherichia coli* strain upon exposure to a low dosage of cefotaxime. The likely mechanism of this action is based on the characteristic of the antibiotics, which inhibit bacterial cell division and trigger the SOS system which is responsible for DNA repair, enhanced mutagenesis, and induction of prophase.^43^ This in-turn can increase the biomass and the biosynthetic capacity of bacterial cells, which virulent phages can take advantage of to increase their own production. Additionally, the presence and effect of these drugs can accelerate the lysis of infected bacteria, which would allow the phages to increase the rate of spread. The drug's effect on targeted biomass seems to be the key component in this PAS response instead of the SOS system.^42^ The PAS phenomenon has shown potential to treat infections by the *Burkholderia cepacia* complex (Bcc), which is a group of at least 18 species of Gram-negative opportunistic pathogens that can cause chronic lung infection in cystic fibrosis patients. Kamal and Dennis reported effectiveness of phage KS12 in the presence of meropenem and minocycline against *Burkholderia cenocepacia* strain K56-2 in *Galleria mellonella* larvae. Infected larvae showed increased survival at 48 hours post infection when treated with a combination of phage and antibiotics compared with the no-antibiotic or no-phage control. The survival rate with phage KS12 and meropenem was 78% compared to phage KS12 or meropenem alone, 33% and 20% respectively. Minocycline had a similar result, with 24% survival alone *versus* 69% survival when minocycline and phage KS12 were combined. Considering treatment of Bcc members is typically very difficult due to their intense resistance to chemical antibiotics, phage therapy may be a potential alternative.^44^

## Designer phages

**Fig. 2:**
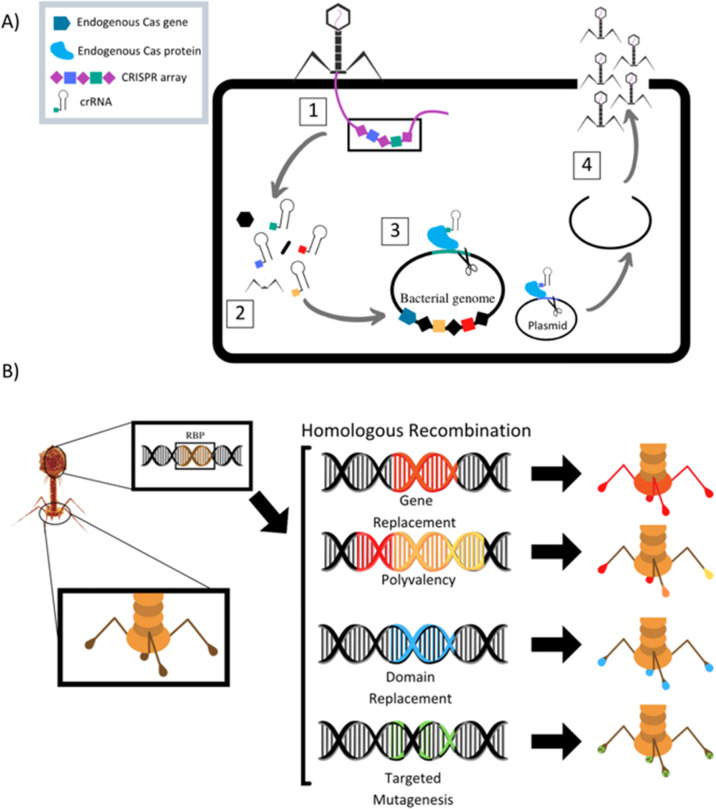
(A) Schematic of homologous recombination of phage receptor binding proteins to alter phage host target range. Modified from Lenneman *et al.* (2021). (B) Schematic of phage triggered CRISPR-Cas9 pathway for activation of endogenous bacterial nuclease *via* CRISPR RNA (crRNA). (1) Phage adsorbs to target bacterium and injects its genome into the cell. (2) Phage enters replication cycle, where spacers contained in the phage genomes CRISPR array are expressed and mature into crRNAs and phage structural proteins are produced. (3) The phage cRNAs guide endogenous Cas9 proteins to cut targeted loci in the bacterial genome and/or plasmids. (4) Cuts lead to cell death if the bacterium is unable to repair genome damage or re-sensitization to antibiotics if antibiotic resistance gene on plasmid is damaged. Phage completes its replication cycle and assembles new progeny virions, which lyse the cell and are released into surroundings. Modified from Fage *et al.* (2021).

The field of synthetic biology has developed designer phages that are genetically modified natural phages that can be used in bacterial diagnostics, therapeutics, and drug delivery. Multiple different methods to modify phage to improve efficacy of antimicrobial action have been designed and reported (Table 1). Phage can be programmed to increase host specificity range, which naturally is very narrow. Phage cocktails containing more than one phage can target various pathogens in an infected system and minimize phage-resistant mutants, but the development and approval process for multiple phages within one treatment is time consuming. To overcome these problems, synthetic biologists are creating adjustable host ranges by altering the phage receptor binding proteins (RBPs).^45^ These RBPs, located at the distal end of the baseplate or at the tip of the tail fibers, mediate the interaction of the phage with host surface receptors. Adjustable host ranges are accomplished by tail fiber exchange *via* homologous recombination and have been effectively created in the closely related T2, T4 and T7 families of phages (Fig. 2). RBPs must be modified carefully, however, as to not lose infectivity properties. An approach has been reported to create chimeric RBPs, by exchanging the globular receptor-binding domains of phage tail fibers, but leaving the N-terminal domain intact, allowing for interactions with the phage tail.^45–49^

**Table 1:** Modulation of phage target or delivery system to effectively target bacterial or fungal (F) infection *in vivo* systems

Study	Phage	Phage target	Phage modification/goal
(A) Norbrega *et al.*	T7	*Escherichia coli*	Non-encapsulation, modification with *E. coli* protein phosphoporin E to resist low pH
(B) Jun *et al.*	Endolysin SAL-1	Antibiotic resistant *Staphylococcus aureus*	Targeting of antibiotic resistant strains *via* IV administration.
(C) Mahichi *et al.*	Modified T2 with IP008 tail fiber sequence	*Escherichia coli*	IP008 tail fiber increases potential target range of T2.
(D) Lu *et al.*	T7 modified to produce Dispersin B	*Escherichia coli* biofilm	Allow for penetration into biofilm.
(E) Yacoby *et al.*	Chloramphenicol linked phage	*Staphylococcus aureus*, *Streptococcus pyogenes*, *Escherichia coli*	Increase antibiotic targeting ability and specificity.
(F) Dong *et al.*	PPA with JM-phage	*Candida albicans*	Deliver a photosensitizer (PPA) to fungus to increase susceptibility.
(G) Schlezinger *et al.*	*E. faecalis* phage in P407	*Enterococcus faecalis*	Injection into infected root canal, P407 serves as biopolymer delivery to help localize phage to site of infection.

Mahichi *et al.* replaced the T2 phage tail fiber genetic sequence with tail fiber sequence of phage IP008, which has a wide-host range. After replication, the host range of the recombinant T2 phage was identical to that of IP008, and lytic activity of the phage against *E. coli* was restored.^50^

Phage can also be designed to deliver genetic “payloads”, which can enhance or modulate phage antimicrobial activity. These payloads typically code for heterologous proteins, such as biofilm-depolymerases, capsule-depolymerases, quorum-quenching enzymes, or cell wall hydrolyases. Upon infection with the genetically engineered phage, cells produce these proteins that are released upon cell lysis and can act on target cells in their vicinity. These payload proteins include CRISPR-Cas9 nucleases, which can effectively create a nucleotide sequence-specific antimicrobial to target host genomes for degradation (Fig. 2).^51^ CRISPR-Cas9 phages allow for selective removal of resistant bacterial sub-populations, and can also prevent antibiotic resistance genes from developing by immunizing the bacterial host against an antibiotic resistance plasmid.^46,52–55^ Phages can be designed to express certain antimicrobial proteins, for example, Lu *et al.* engineered T7 phages to produce the biofilm degrading enzyme, Dispersin B. When phage replicated within *E. coli* microbes contained in a biofilm, lysis and release of progeny lead to effective biofilm disruption and increased ability of phage to diffuse into the biofilm matrix, promoting infection clearance.^56^

Not only can phage deliver genetic payloads to target microbes, but additional drugs can also be physically conjugated to the surface proteins of phage capsules to allow delivery, and synergy between phage treatment and antibiotics can improve efficacy of both. For example, Yacoby *et al.* connected hydrophobic drugs to phage capsule proteins *via* aminoglycoside antibiotics which served as solubility-enhancing bridges.^57^ This greatly increased carrying capacity of the phage, demonstrated through complete growth inhibition of *Staphylococcus aureus*, *Streptococcus pyogenes*, and *Escherichia coli* using chloramphenicol linked to the phage through the aminoglycoside neomycin. The aminoglycoside linkage allowed an increase of carrying capacity from less than 3000 drug molecules/phage to ∼11 400 drug molecules of chloramphenicol per phage.^57^ Phage engineering can be used against opportunistic fungal infections as well, as shown by Dong *et al.* who conjugated pheophorbide A (PPA) with a JM-phage using the 1-ethyl-3-(3-dimethylaminopropyl)-carbodiimide hydrochloride (EDC)-*N*-hydroxysuccinimide (NHS) method for light irradiation against *Candida albicans.*^58^ PPA is a chlorophyll-based photosensitizer that is localized in the mitochondria and activated by 670 nm wavelength of light, with high phototoxicity and low efficiency without assisted targeting. JM-PPA conjugated phage was able to effectively infect *C. albicans* and induce shrinking and rupturing of fungal cells upon light activation of the PPA photosensitizer.^58^

One major concern with phage therapies is host immune response to released cytotoxic cellular components following lysis of bacterial host by phage. A potential solution to this problem relies on non-lytic (lysogenic) phage and non-replicating protein capsules, or phagemids. These second-generation phage therapeutics involve delivery of genetic networks to help eliminate bacterial targets using non-lytic means. For example, antibiotic susceptibility factors can be delivered *via* phage, which can leave target bacterial populations hyper-sensitive to the standard mechanisms of antibiotics. Certain phages have been developed to overexpress transcriptional factors that repress the SOS response and DNA repair, which leads to increased susceptibility to antibiotics upon phage infection.^59^ Phages have also been engineered to induce microbial expression of dominant-sensitive genes, that result in antibiotic re-sensitization, effectively reversing antibiotic resistance.^1^ Additional mechanisms include phage induced expression of small regulatory RNAs in bacterial hosts, which knock down the expression of resistance genes, recovering antibiotic susceptibility.^60^

## Controlled delivery strategies *via* biomaterials

**Fig. 3:**
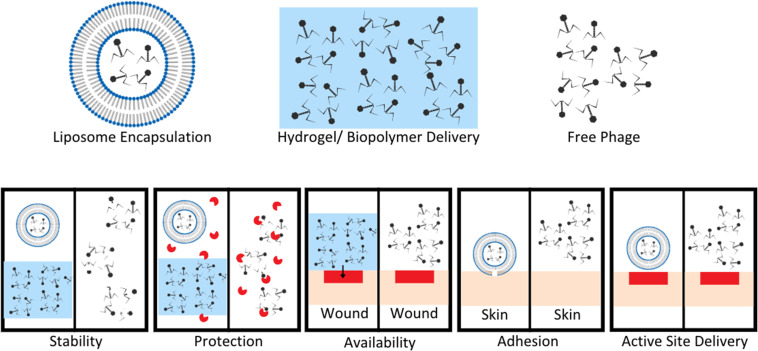
Benefits of encapsulation or hydrogel delivery systems compared to free phage delivery. Stability of phage improved by encapsulation in liposomes or hydrogel to limit exposure to extreme pH and other degradation factors. Protection from enzymes increases with liposome encapsulation and hydrogel suspension compared to free phage easily targeted and degraded by enzymes. Hydrogel application to wound sites allows for localization of phage to a wound bed compared to free phage application. This leads to increased concentration of phage at wound sites, and phage titer is a known to enhance phage therapy efficacy. Adhesion and active site delivery can be facilitated by liposome encapsulation to improve direct interaction with target tissues. Modified from Loh *et al.* Appl. Environ. Microbiol. (2021, **87**, e01979–20, DOI: https://doi.org/10.1128/AEM.01979-20, amended with permission from American Society for Microbiology).

Phage therapies benefit from modified controllable delivery strategies over free phage delivery in a variety of ways, included stability, localized availability, protection from enzymatic degradation, attachment, and active site delivery (Fig. 3).^61–63^ Upon development and standardization of phage therapies, biomaterial encapsulation of bacteriophages will need to be developed to improve storage stability, to avoid the need for a cold supply chain, and to allow phages to survive production stresses and remain viable degradation is required, whereas intravascular injected longer to improve length of dosing.^27^ Encapsulation goals vary based on method of administration for phage therapies (Fig. 3).^61^ For orally administered phage, protection from gastric activity and enzymatic administration could result in short half-lives. Encapsulation in nanovesicles, for example pegylated liposomes, is a potential solution to multiple problems. Liposome encapsulation can reduce phage interaction with biofilm surface proteins due to the specific binding nature of their proteins to bacterial surface targets and not EPS proteins, allowing improved diffusion into bacterial targets within biofilms.^62^ Liposome encapsulation of phage also can increase stability in the gastric environment and have been shown to adhere to intestinal membranes of chickens with penetration through the mucosal layer to target infectious bacteria.^62,63^ Stability of phages UAB_Phi20, UAB_Phi78, and UAB_Phi87 in a 1 : 1 : 1 cocktail ratio encapsulated in cationic liposomes was tested in the ceca of 126 broiler chickens over 72 hours (63 with non-encapsulated and 63 with encapsulated phages). The presence of phages in the cecum of each animal was determined at 2, 48, and 72 hours with encapsulated groups having significantly higher percentages of broilers with phage still present in their ceca after 48 and 72 hours. This highlights the necessity for encapsulation or modification to maintain phage concentrations and viability in gastrointestinal environments.^63^

Controlled release of phage therapies can also be accomplished *via* encapsulation in biomaterials. Incorporation of phage-loaded biopolymers into surgical implants or introduced during the surgical procedure could limit need for repeat administration, assuming phage stability is acceptable. Potential biopolymers currently studied include hydrogels derived from collagen, fibrin, agarose, and alginate. Synthetic polymers and semisynthetic polymers have also been reported, included polyethylene glycol (PEG), polyacrylamide (PA), and polyvinyl alcohol (PVA).^64^ Stimuli responsive controlled release can be triggered by multiple variables, including enzymes, pH fluctuations, temperature fluctuations, exposure to light, or hydrolytic conditions.^65,66^ Shlezinger *et al.* reported a temperature trigger sustained release of *Enterococcus faecalis* phage encapsulated in the poloxamer P407 which is liquid at 4 °C but gels at room temperature. The phage was encapsulated at a 30% w/v to P407 liquid solution and injected into infected root canal models in rats, resulting in significant reduction in bacterial counts, 3 weeks after treatment with phage. Sustained release was confirmed over a one month period in PBS buffer.^67^ One potential problem of phage-delivered biomaterials lies in sterilization of phage-containing biomaterials, considering phages are vulnerable to almost all commonly used sterilization processes, included steam sterilization, ethylene oxide treatment, and gamma radiation.^66^ Therefore, phage-incorporating biomaterials must be processed sterilely prior to phage addition, which can be costly and time-consuming. To maintain integrity and sterility of the phage and encapsulating materials, these materials must be prepared and stored properly to maximize shelf life.

## Future treatment avenues

Therapies utilizing bacteriophage have potential for combatting a variety of human bacterial infections, especially in combination with antibiotic regimens. Treatment of chronically infected epidermal wounds is one possibility, which often occur in immunocompromised and diabetic individuals. Phage treatment can be administered *via* topical solutions, injection into or around the wound, or *via* biodegradable biopolymer would dressings with time-release abilities. Intracellular infections (*Mycobacterium* spp.) need to be explored further to develop effective modes of bacteriophage delivery into mammalian cells, although pathogens that have a temporary extracellular living phase can be more easily targeted by phages. One example is that of *Mycobacterium ulcerans*, which Trigo *et al.* has shown to be effectively targeted by phage therapy in a murine footpad model.^68^ In the case of antibiotic-resistant strains, Golkar *et al.* described effectiveness of phage PS5 against a multi-drug resistant strain of *Pseudomonas aeruginosa* in mouse models which specifically focus on efficacy of this treatment.^69^ Mice were injected twice with lytic PS5, once 30 minutes after bacterial infection, and again 24 hours later, and then given a daily dose of PS5 orally *via* drinking water following. Complete resolution of wound models was seen after 6 days in phage treated groups as compared to remaining open lesions on the backs of infected non-treated animals. Both intraperitoneal and oral administration was determined to be effective modes of treatment.^69^

Mucosal membranes are of particular interest in treating bacterial colonization with phage therapies, particularly due to the severity of infections. Within respiratory, gastrointestinal, urogenital infection phage therapy, *Klebsiella pneumonia*, *Acinetobacter baumannii*, *Pseudomonas aeruginosa*, and *Staphylococcus aureus* have been studied extensively. However, there are still a multitude of pathogens still to be explored in treatment with phage therapies, including upper respiratory infections of *Haemophilus influenzae*, *Corynebacterium diphtheria*, and *Porphyromonas* species, lower respiratory infections of *Helicobacter pylori*, *Mycobacterium simiae*, or *Nocardia* species. Etiological species of pneumonia infections including *Mycoplasma pneumonia*, *Fluoribacter bozemanae*, or *Ureaplasma urealyticum* also present a significant group for potential applications of phage therapy.^70^ Respiratory infections would be most effectively treated *via* inhalant administration for direct routes into respiratory tracts and to minimize exposure of other patient systems to phage. For GI infections, when orally administered, phage encapsulation or modulation is key in avoiding sensitivity to the low pH and digestive enzymes of the gastrointestinal tract. Effective treatment of *Escherichia coli* has been shown in feedlot cattle with *E. coli* using O157:H7 phages.^71^ Phage therapy also offers advantages over traditional antibiotic treatments by limiting disruption of the natural gut flora. Treatment of urogenital tract infections needs further research, as there are no known lytic phages for *Legionella pneumophila*, *Proteus penneri*, *P. rettgeri*, *Citrobacter koseri* species. Additionally, phage therapy offers strong potential for the treatment of the causes of sexually transmitted infections, including *Haemophilus ducreyi* (chancroid), *Chlamydia trachomatis* (chlamydia), *Neisseria gonorrhoeae* (gonorrhea), *Treponema pallidum* (syphilis), and *Klebsiella granulomatis* (granuloma inguinale).^72,73^

Finally, phage therapies show potential for addressing life threating system infections, which can easily develop from common localized infections, such as pneumonia, urinary tract, or bladder and skin infections. Pathogens of specific concern include: *Francisella* species, *Letospira interrogans*, *Brucella canis*, *Ehlichia sennetsu*, *Reckettsia* species, *Treponema* species, *Peptostreptococcus anaerobius*, *Clostridium tentani*, and *Clostridium botulinum*. Treatments *via* IV administrations could have potential to clear infection from the bloodstream, and transdermal delivery could offer another treatment avenue.^70^ For systemic treatments, this can lower risk of damage to host tissues which can occur with chemical antibiotic treatments, since phage are only infecting specifically targeted bacteria, which can help preserve both tissue integrity of the patient and their natural microbiome.

Phage therapies have advantages over current bactericidal treatment regimens, specifically their ability to be modified, their specificity, and current lack of resistant hosts. Purification methods of phage populations for therapies must be developed and continually improved in consistent and repeatable methods, and continued testing of stability, sterility, and functionality must be completed to ensure efficacy of treatments.^74^ Problems to address include the lack of established rules for quality control of potential phage therapies, as strenuous testing is needed to guarantee safety and effectiveness of phage as antibacterial or other targeted therapies.

## Conclusions

As phage therapies are shown to be safe and effective, a phage treatment will become more commonplace for infections, possibly alone or in combination with existing antibiotics. Phage therapy offers an avenue to reduce antibiotic resistance against commonly used treatment regimens, allowing continued use of currently available antibiotics. Engineering of bacteriophage genomes increases target ranges and genetic payload delivery to bacterial culture, while remaining specific to particular host targets. This specificity will reduce impact to natural microbiome cultures, leaving healthy host systems intact while targeting potential pathogens *in vivo*. Engineering of encapsulation or modification of phage to increase stability and storage capabilities will continue to allow larger, more controllable, and predictable volumes of phage to be administered into host systems for treatment, focusing on materials that are hydrophilic, able to be synthesized under mild environments, non-toxic, and environmentally friendly. New controlled delivery approaches could also allow for stimuli responsive release and tailoring of release profiles. A continuation of studies both *in vitro* and clinical trials is necessary to study safety and efficacy of treatments, before phage therapeutics can become more commonly used for treatment of infection in humans. Guidelines need to be established as well to determine most effective mode of delivery for different types and locations of infections, which also demands establishment of effective infection models, especially for chronic and multidrug resistant varieties.

## Conflicts of interest

There are no conflicts to declare.

## Supplementary Material
